# There Is No Simple Model of the Plasma Membrane Organization

**DOI:** 10.3389/fcell.2016.00106

**Published:** 2016-09-29

**Authors:** Jorge Bernardino de la Serna, Gerhard J. Schütz, Christian Eggeling, Marek Cebecauer

**Affiliations:** ^1^Science and Technology Facilities Council, Rutherford Appleton Laboratory, Central Laser Facility, Research Complex at HarwellHarwell, UK; ^2^Institute of Applied Physics, Technische Universität WienWien, Austria; ^3^MRC Human Immunology Unit, Weatherall Institute of Molecular Medicine, University of OxfordHeadley Way, UK; ^4^Department of Biophysical Chemistry, J.Heyrovsky Institute of Physical Chemistry, Czech Academy of SciencesPrague, Czech Republic

**Keywords:** plasma membrane, membrane organization models, nanodomains, heterogenous distribution, membrane physical properties

## Abstract

Ever since technologies enabled the characterization of eukaryotic plasma membranes, heterogeneities in the distributions of its constituents were observed. Over the years this led to the proposal of various models describing the plasma membrane organization such as lipid shells, picket-and-fences, lipid rafts, or protein islands, as addressed in numerous publications and reviews. Instead of emphasizing on one model we in this review give a brief overview over current models and highlight how current experimental work in one or the other way do not support the existence of a single overarching model. Instead, we highlight the vast variety of membrane properties and components, their influences and impacts. We believe that highlighting such controversial discoveries will stimulate unbiased research on plasma membrane organization and functionality, leading to a better understanding of this essential cellular structure.

Membranes are one of the key structures in cell biology. Besides being instrumental in compartmentalizing and protecting cells, their role as organizing centers for tasks such as metabolism or signaling is increasingly recognized. In fact, a majority of cellular processes are associated with membranes (Stryer, 1995). Membranes provide useful docks for correct localisation of proteins which is essential for their function (Miosge and Zamoyska, 2007; Grecco et al., 2011; Hung and Link, 2011). Importantly, in humans, mislocalization of membrane proteins leads to the loss-of-function and, frequently, can develop into diseases (Edwards et al., 2000; Matsuda et al., 2008; Hung and Link, 2011; Schaeffer et al., 2014). Nevertheless, the presence of proteins at a particular membrane is usually not sufficient for their function. Often, the nanoscopic localization, oligomerisation and/or clustering of membrane proteins can affect the efficiency of cellular processes (Cebecauer et al., 2010; Matthews, 2012; Nussinov, 2013; Garcia-Parajo et al., 2014). Membranes, the lipid environment and membrane properties in general, influence nanoscale organization and function of these molecules. It is, therefore, important to understand molecular details of membrane structure and mechanisms responsible for its dynamics organization.

Here, we review membrane properties, models of membrane organization and useful techniques for studies of membrane organization and dynamics, with a special focus on the plasma membrane of higher eukaryotes (mammals). Our specific aim is to re-emphasize currently omitted or underestimated biophysical principles and discuss their role in dynamic membrane organization. We attempt to provide a comprehensive description of membrane complexity and suggestions how to avoid interpretation of membrane-associated phenomena within the borders of a single theory. As a reader will see, we believe that there is no universal model of the plasma membrane dynamic lateral organization. These more general issues will be discussed in the last section. First, let us start with the very basic structure of membranes.

## Basic structure of cell membranes

**Figure 1:**
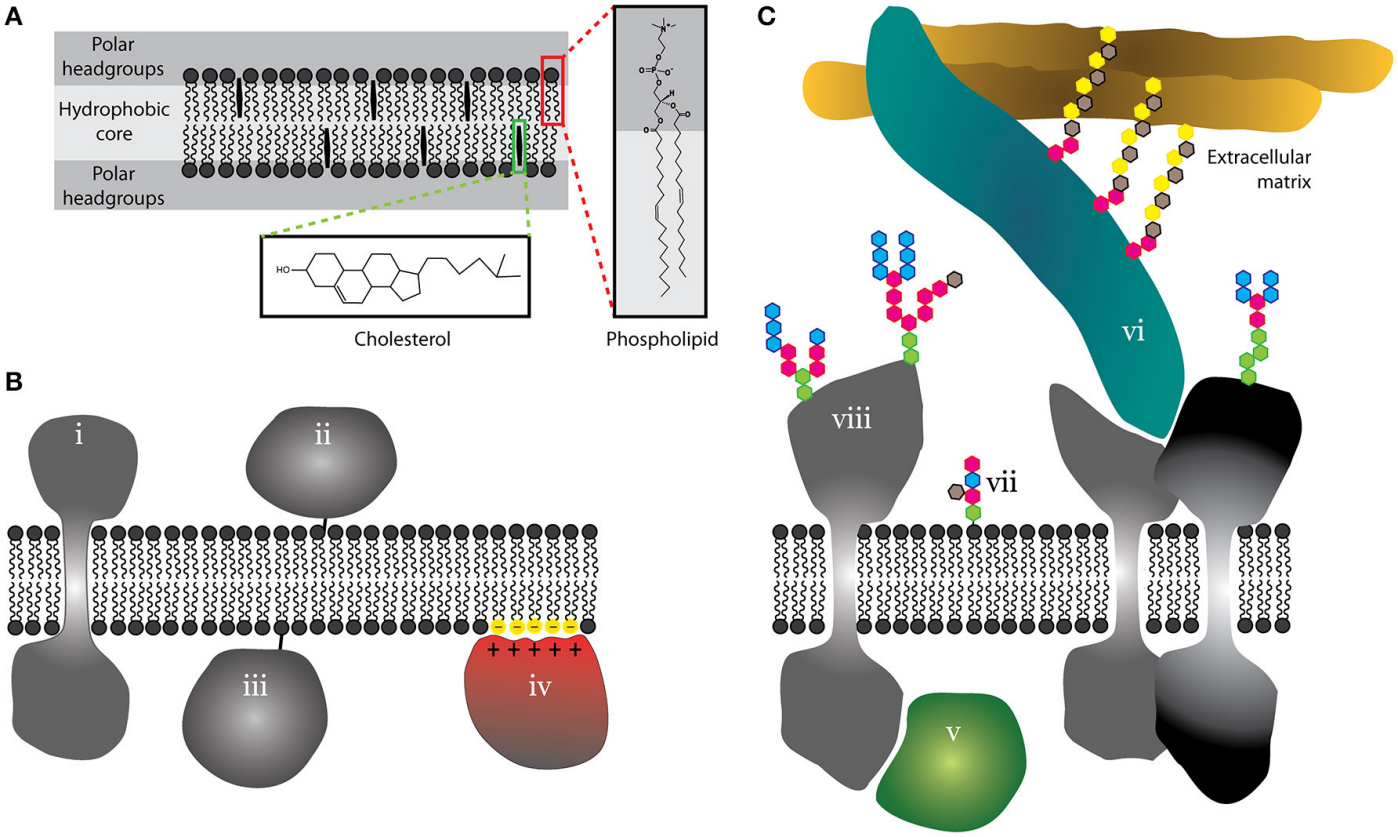
**Schematic illustration of the basic structure of lipid bilayer and proteo-lipidic membranes. (A)** Cell membranes are lamellar structures with a hydrophobic core and a polar headgroup space. As examples, phospholipids and cholesterol are shown with almost atomistic detail (red and green boxes). **(B)** Membrane proteins can integrate into membranes (i), but can use lipid anchors (ii and iii) or peripherally associate with membranes via electrostatic interactions (iv). **(C)** Proteins can further associate with membranes via protein-protein interactions on the cytosolic side (v) or at the interface between the plasma membrane and the extracellular matrix (vi). Outer leaflet lipids (vii) and extracellular domains of proteins (viii) are often glycosylated.

A lipid bilayer forms the basis of all cellular membranes. It is a lamellar structure with a hydrophobic core and a polar headgroup region on both sides (Figure 1A). In cells, it is composed of hundreds, if not thousands, of different phospholipid species. These differ in their polar headgroup moiety but mainly in the length and saturation of acyl chains forming a hydrophobic core of a lipid bilayer. Other lipid and fatty acid species add to this complexity. Of those, sterols (cholesterol in mammals) are the most abundant in the plasma membrane and can represent up to 40% of total lipid (van Meer and de Kroon, 2011). Cholesterol has a special structure (Figure 1A) enabling strong impact on basic membrane properties such as viscosity or interleaflet coupling, as described multiple times in comprehensive articles (Ipsen et al., 1987; Mouritsen and Zuckermann, 2004; Maxfield and van Meer, 2010).

Proteins constitute approximately half of the total plasma membrane mass (Dupuy and Engelman, 2008). We distinguish integral and peripheral membrane proteins depending on their anchorage into a lipid bilayer via transmembrane domain(s) or a lipid moiety, respectively (Figure 1B). In addition, some proteins may associate with the membrane via electrostatic interactions with lipid headgroups (Figure 1B; McLaughlin and Murray, 2005) or a variety of protein-protein or protein-glycan interactions (Figure 1C; Stryer, 1995). Such proteins are commonly termed as “membrane-associated.” Extracellular parts of lipids and proteins are frequently glycosylated (Figure 1C). Indeed, glycans form a dense structure at the outer surface of the plasma membrane (Berrier and Yamada, 2007). This molecular complexity of membranes has probably evolved to serve as a selective barrier and organizing center with a high fidelity and robustness (Cebecauer et al., 2010). But what are those unique properties which were selected in the process of evolution to control critical cellular processes with such efficiency?

## Intrinsic properties of cell membranes essential for their function

Early definitions, of which the “fluid mosaic model” of Singer and Nicolson (SN model Singer and Nicolson, 1971, 1972) is the best known, highlighted **fluidity** as one of the most critical membrane features. Indeed, fluidity of membranes provides important advantage over other cellular structures such as the cytoskeleton or ribonucleoproteins. It forms the basis for the highly dynamic character of membrane-associated (bio)chemical reactions and other cellular processes. Membrane fluidity enables the majority of molecules to diffuse freely over long distances and rotate or re-orientate to adopt optimal conformation. Membranes can be considered as two-dimensional solutions. This two-dimensional character also distinguishes membranes from other three-dimensional cellular solutes (e.g., the cytosol). The fundamental importance of fluidity is, for example, underlined by the fact that cells modify the saturation of their lipid acyl-chains to keep their membranes fluid when adapting to the environment, e.g., different temperatures (Fraenkel and Hopf, 1940; Buda et al., 1994).

Although membranes are fluid, they have higher **viscosity** (Box 1) than the cytosol (Luby-Phelps et al., 1993). This has a direct impact on the mobility of membrane molecules. Membrane viscosity can be modified by lipid composition or other factors, such as the presence of proteins or poorly mobile structures, and will thus vary over space and time.

Box 1 **Membrane fluidity, viscosity and mobility**

Viscosity is a macroscopic parameter describing the behavior of a large, rigid sphere in a Newtonian fluid. Its use for membranes is imperfect and should be treated with care (Valeur and Berberan-Santos, 2012; Olšinová et al., 2014). Membranes are nanoscopic structures with 2D character and highly heterogeneous composition in terms of size and chemistry. Due to a lack of a better parameter, we use the term “viscosity” to describe membrane properties such as membrane lateral compressibility and acyl chain ordering, which influence the mobility of membrane components. Other terms, e.g., “microviscosity” or “rigidity” were also used in literature to cover these properties in one word (Shinitzky and Inbar, 1976; Kowalska and Cierniewski, 1983; Gut et al., 1985; Sherbet, 1989).

The term “fluidity” is frequently used to replace “viscosity” for biological membranes or other highly heterogeneous materials (Valeur and Berberan-Santos, 2012). We use term “fluidity” in this work to distinguish membranes from other cellular structures which exhibit much higher stability (e.g., nucleoproteins), thereby limiting rapid, long-range mobility of associated compounds.

Efforts to measure viscosity of cellular membranes are associated with serious technical difficulties (Valeur and Berberan-Santos, 2012; Olšinová et al., 2014). Instead, measurements of rotational or lateral diffusion were successfully applied to characterize membrane viscosity. In cellular membranes, lateral diffusion is frequently substituted with the term “mobility.” Mobility of membrane molecules can be influenced by many different factors, such as (i) membrane ordering or, in the other terms, how densely lipids and proteins are packed in the membrane (Kahya et al., 2003), (ii) lateral pressure of the membrane which is partially linked to ordering but also membrane hydration (polarity) and directly influences bilayer compressibility and elasticity (Marsh, 1996; Cantor, 1999), and (iii) macromolecular crowding (Saxton, 1987; Guigas and Weiss, 2015). Mobility of membrane components is further influenced by other intrinsic and extrinsic factors as described in the main text.

To illustrate dramatic differences in the mobility of molecules in synthetic and cellular environments, we provide a few values of diffusion coefficients in Table 1. These should be considered as a simple guideline due to differences in the precision with which these values were measured. We also provide the time scale a molecule requires to traverse the distance of 20 μm (longitudal size of HeLa cells) by random (Brownian) 2D motion. This should underline dramatic differences in the mobility of molecules in real space.

Another property emphasized in the SN model is **continuity** of the plasma membrane (Singer and Nicolson, 1972). The plasma membrane fully covers the cell surface. Its continuity is especially important for membrane receptors or effector molecules which need rapidly to re-localize, e.g., when a cell is changing its direction of chemotactic mobility (Janetopoulos and Firtel, 2008). Continuity also supports intermolecular interactions or the formation of multi-molecular assemblies within or at the surface of membranes. In some cells, membrane continuity is limited to the apical or basal side due to the presence of tight junctions eliminating free mobility of membrane molecules (Balda and Matter, 2008). We will discuss viscosity and continuity, and their impact on the organization of membranes in more detail further in the text.

Almost all molecules can interact and influence each other in cellular membranes. As a consequence, coexistence of molecules in membranes has **cooperative** character. Cooperativity of molecules was already mentioned for fluid cellular membranes in the SN model (Singer and Nicolson, 1972) but seems to be recently overseen. This property has a dramatic impact on experiments, in which systemic perturbance of membranes (e.g., by chemical or genetic treatment) was employed to support specific models of membrane organization.

**Figure 2:**
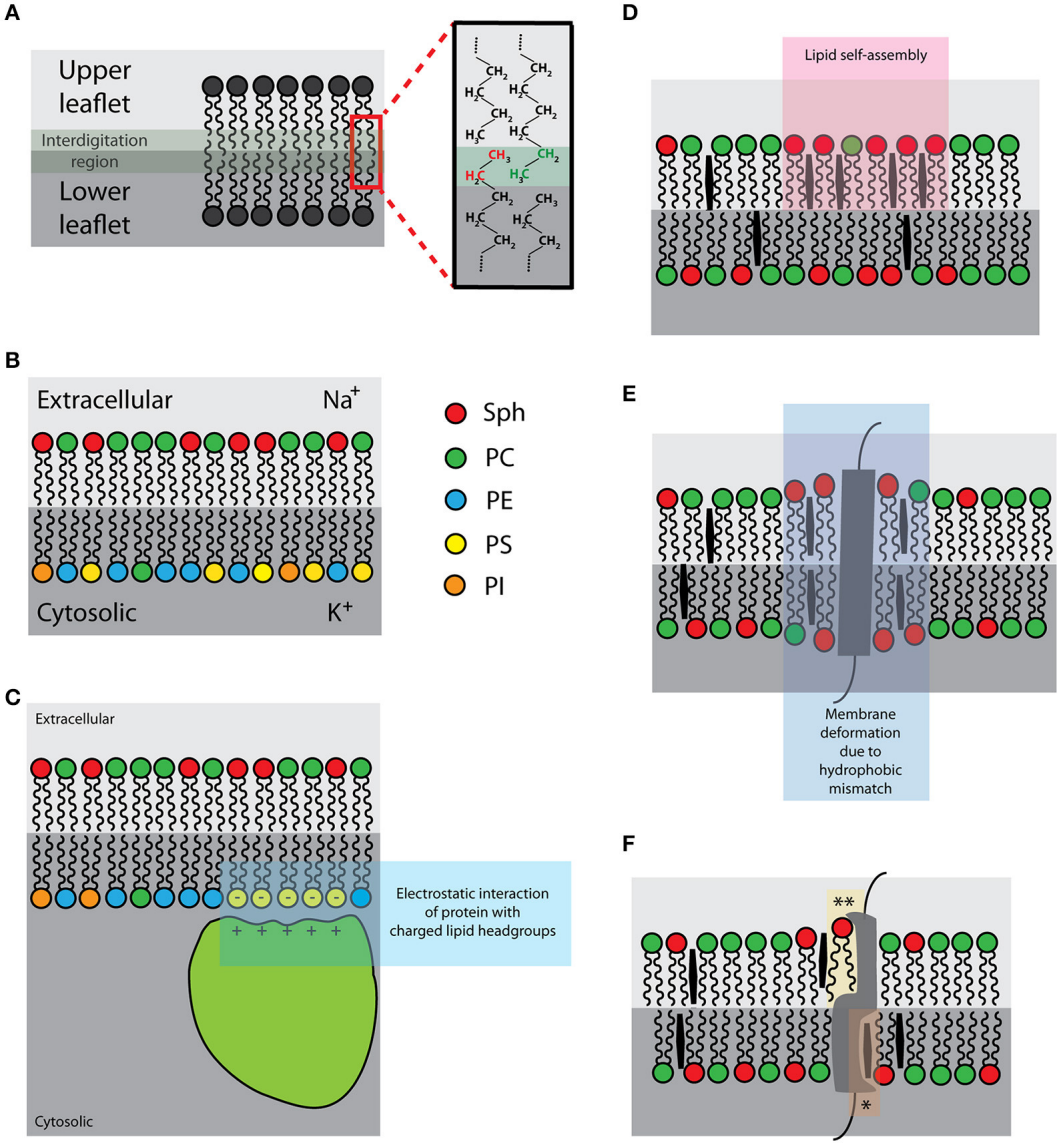
**Schematic illustrations of the selected intrinsic membrane properties: (A)** Interleaflet coupling [interdigitating lipid acyl chains in green-gray; zoom: interdigitating ethyl groups of upper (green) and lower (red) leaflets]; **(B)** Asymmetric distribution of lipids and ions [right hand-side: color-coding of lipid species]; **(C)** Negatively charged lipids (yellow) of the plasma membrane inner leaflet [for the association of proteins with basic-rich domains (light green)]; **(D)** Lipid self-assemblies (pink); **(E)** Hydrophobic mismatch (purple); **(F)** Protein-lipid interactions [^*^sphingolipid- and ^**^cholesterol-binding pockets].

Lipid membranes undergo **interleaflet coupling**, meaning that acyl chains of lipids in one leaflet interdigitate into the space of the other leaflet (Figure 2A; Nickels et al., 2015). Theoretical predictions suggest that interleaflet coupling can coordinate the organization of molecules between the two leaflets (Schmidt et al., 1978; Duzgunes et al., 1988; Merkel et al., 1989; Kiessling et al., 2006; Raghupathy et al., 2015; Williamson and Olmsted, 2015). Yet, White and co-workers recently provided an alternative view (Mihailescu et al., 2011; Capponi et al., 2016). They do not negate the existence of strong coupling between the two leaflets of lipid bilayer but observed no direct complementarity between the opposite acyl chains (Capponi et al., 2016). Cholesterol, which was predicted to intensify interleaflet coupling in membrane lipid domains, was found to reduce the level of acyl-chain interdigitation (Mihailescu et al., 2011). These works indicate that we need more experimental data in order to better understand the effect of interleaflet coupling in lipid bilayers.

The plasma membrane of eukaryotic cells is **asymmetric** (Figure 2B) in terms of lipid and surface ion composition, as well as the presence of specific proteins (Rothman and Lenard, 1977; van Meer et al., 2008). The lipid asymmetry is maintained by flippases and other lipid translocating or transport proteins (Canagarajah et al., 2008; Devaux et al., 2008). Chemical asymmetry, a gradient of ions, drives a number of vital cellular processes (e.g., generation of chemical energy and metabolism). On the other hand, lipid asymmetry further adds to the diversity and complexity of cellular compartments, thereby helping to optimize cellular processes. For example, negatively charged lipid headgroups in the inner leaflet provide the binding surface for proteins with specific binding domains (Figure 2C; McLaughlin and Murray, 2005). This can cause protein relocalisation often leading to the initiation of signaling events (Yeung et al., 2008). In addition, chemical asymmetry and the presence of ions induces heterogeneous distribution of lipids, at least in simulations and in model systems (Vácha et al., 2009; Jurkiewicz et al., 2012). Whether this effect contributes to the organization of plasma membrane in living cells is experimentally difficult to test; an asymmetric membrane is indispensable for cell viability. At the same time, the formation of asymmetric model membranes *in vitro* is a rather delicate process and was successfully performed only in a few cases in past (Kiessling et al., 2006; Collins and Keller, 2008; Chiantia et al., 2011). Therefore, data demonstrating lateral (re)organization due to membrane asymmetry are still rare.

Even though lipids interact only weakly, preferential **self-assemblies** of certain lipid species or conformations (Figure 2D) were demonstrated in model lipid mixtures (Björkbom et al., 2010; Ivankin et al., 2010). Under certain circumstances, lipid self-assembling may extensively reduce miscibility of its molecules, i.e., generate physico-chemical heterogeneities. A well-known example of lipid self-assembly and segregation is the formation of separated lipid phases in vesicles composed of two or more lipid species with different melting points (Bagatolli and Gratton, 1999; Korlach et al., 1999; Bernardino de la Serna et al., 2004; Veatch and Keller, 2005). Importantly, lipids are prone to phase separation or miscibility transitions also in cell membrane-derived vesicles and blebs, as well as artificial vesicles generated from lipids extracts and from native membranes (Bernardino de la Serna et al., 2004; Baumgart et al., 2007; Veatch et al., 2008). All these observations were achieved using equilibrated membranes; however, cells are non-equilibrium systems (Stryer, 1995). Indeed, no miscibility phase transitions were observed in living cells over a wide range of temperatures (Lee et al., 2015). Putative impact of lipid self-assembly and ordered lipid membranes on cell membranes is discussed in the section “Plasma membrane organization–general models and concepts.”

Hydrophobic thickness of a lipid bilayer is defined mainly by the length and saturation of acyl chains and the presence of sterols. Bilayer lipids interact non-specifically and transiently with transmembrane domains of integral proteins (Marsh, 1993). Imparity of the hydrophobic thickness of the bilayer and the hydrophobic length of TMD (s) is called **hydrophobic mismatch** (Figure 2E). Hydrophobic mismatch was proposed to induce molecular aggregation/segregation in lipid bilayers, as described in the mattress model (Mouritsen and Bloom, 1984). For example, lipids with longer and more saturated acyl chains will preferentially reside in the annulus of helical TMD with long hydrophobic length. More about the mattress model is discussed in the section “Plasma membrane organization–general models and concepts.”

Lipids can also interact with proteins in a more specific manner (Haberkant et al., 2008; Fantini and Barrantes, 2013; Yeagle, 2014). Several proteins carry lipid-binding domains (Ernst et al., 2010; Contreras et al., 2011; Fantini and Barrantes, 2013) to which lipids bind with a higher affinity compared to the lipids of the first shell interacting with transmembrane domains non-specifically. Such **protein-lipid interactions** (Figure 2F) can be highly specific in a way that lipid headgroup, acyl chain length and its saturation determine the affinity of such interactions (Contreras et al., 2012). Specific protein-lipid interactions have been shown to modulate protein stability and its function (Uittenbogaard and Smart, 2000; Hanson et al., 2008; Contreras et al., 2012) or are directly involved in transport of lipids between subcellular compartments (Kwon et al., 2009). But what is their impact on the lateral organization of plasma membrane is to date unclear.

The abovementioned intrinsic properties can be ascribed to any proteo-lipid membranes, independent of whether these are artificial or cellular structures. But what is so specific about membranes of living cells? Can “clever” use of these intrinsic properties, their local amplification, reduction and/or combination lead to such limitless concert of events such as metabolism and signal transduction? Or is there a need for extrinsic factors to support those basal membrane properties?

## Extrinsic factors influencing the plasma membrane organisation

The plasma membrane is built to interact with surrounding structures such as cortical actin, the extracellular matrix or a variety of ligand molecules. These form the basis of extrinsic factors which can shape the plasma membrane.

We assigned **protein-protein interactions** to the section of extrinsic factors, given the fact that extra-membranous (extracellular and cytosolic) domains are the predominant structures involved in persistent associations of proteins. Further, since these interactions often involve non-membranous protein scaffolds, we believe that protein-protein interactions have, to some extent, extrinsic character.

**Figure 3:**
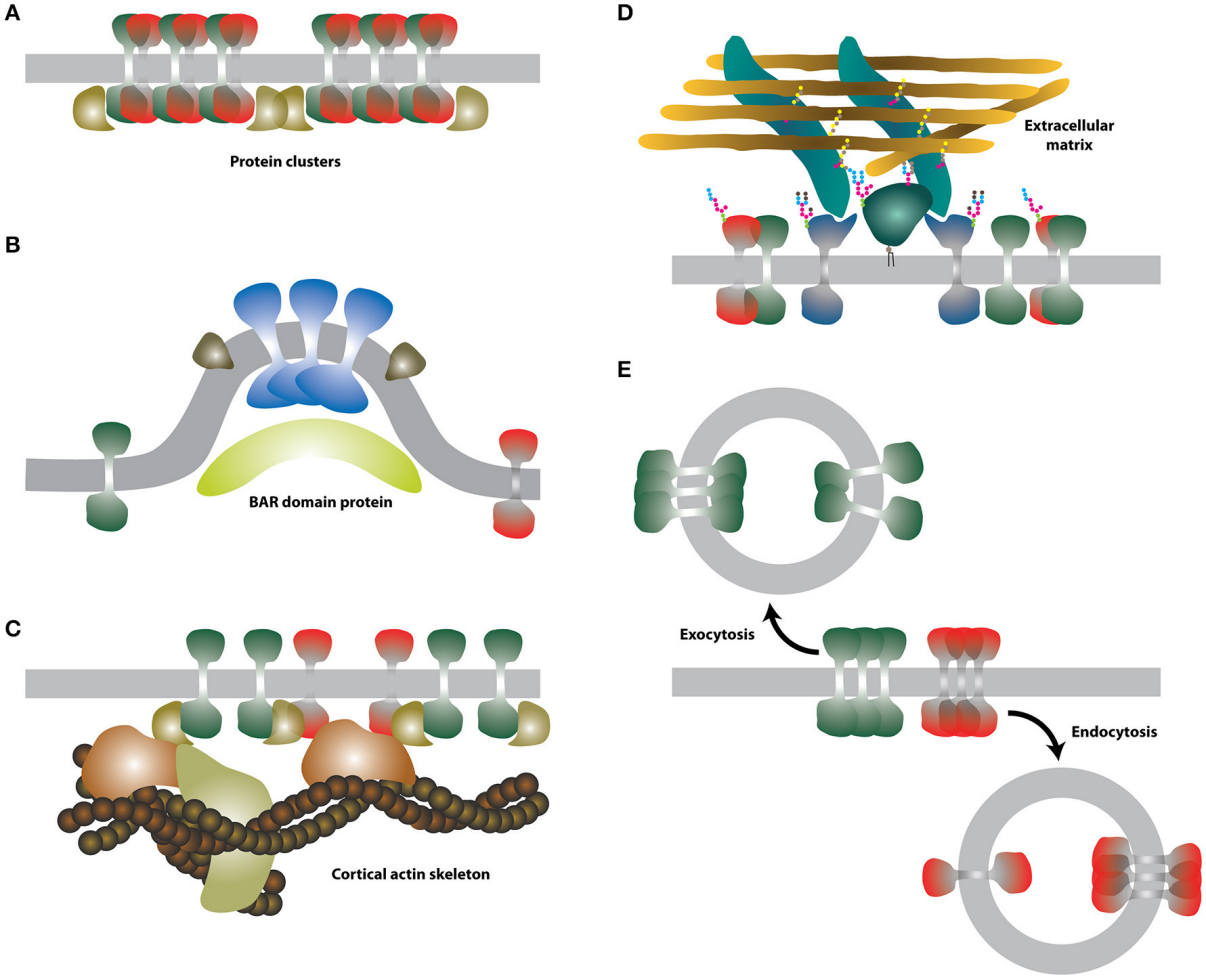
**Schematic illustrations of the selected extrinsic membrane properties. (A)** Protein-protein interactions. Putative membrane proteins (green and red) forming heterodimers can assemble into larger clusters and be stabilized by further interaction with cytosolic proteins (dark yellow). **(B)** Membrane curvature. Certain proteins (blue and green-brown) may prefer curved membranes. Curved membranes can be stabilized e.g., by BAR proteins (light yellow). **(C)** Intracellular cortical actin skeleton. Actin-binding proteins (dark yellow and orange) can associate with integral membrane proteins and form larger assemblies with reduced mobility. **(D)** Extracellular glycocalyx. Interaction of certain membrane proteins (dark blue and dark green) with the extracellular matrix may lead to the formation of larger assemblies with reduced mobility. **(E)** Endo-/exocytosis. Membrane lipids and proteins are rapidly endocytosed (red) or exocytosed (green).

In contrast to lipids, proteins can interact with high affinity and thus form relatively stable structures (Figure 3A) within a sea of lipid molecules. Indeed, the interaction of proteins is a common process associated, for example, with leukocyte signaling or cellular adhesion, both taking place at the surface of cells (Douglass and Vale, 2005; Rossier et al., 2012). Supramolecular complexes of proteins can be relatively large and can further interact with other cellular components such as the cytoskeleton, thereby forming protein networks which can have local or systemic impact on membranes (see below).

**Figure 4:**
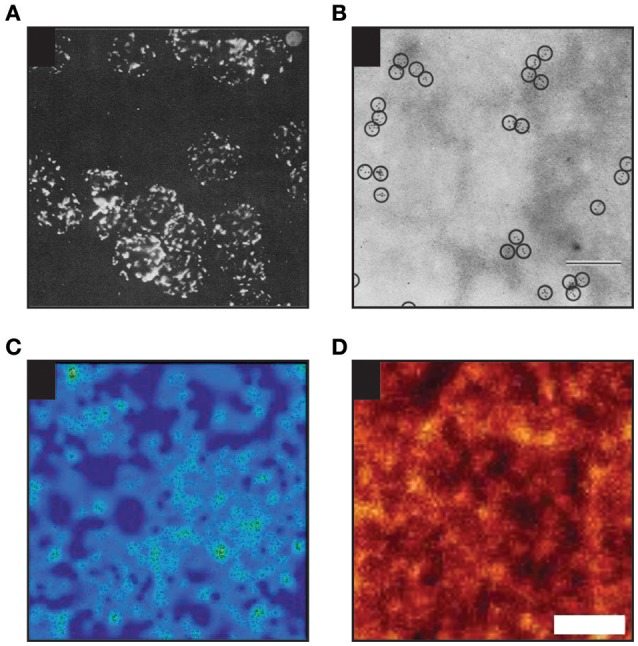
**Examples of heterogenous distribution of proteins in the plasma membrane of mammalian cells. (A)** Heterogenous distribution of MHC molecules as observed on murine lymphoid cells in 1967 by Cerottini and Brunner using epifluorescence microscopy (Adapted by permission from John Wiley and Sons Ltd; (Cerottini and Brunner, 1967). **(B)** MHC clustering on murine red blood cells as detected in 1971 by Nicolson and colleagues by electron microscopy (EM; scale bar: 200 nm; Adapted by permission from RUPress: ©1971 Nicolson et al., 1971). **(C)** Distribution of individual TCR molecules on activated primary human T cells analyzed by dSTORM (Adapted by permission from Macmillan Publishers Ltd: Nature Methods (Rubin-Delanchy et al., 2015), copyright 2015). Showing 3 × 3 μm area. **(D)** Distribution of proteins in membrane sheets derived from a neuroendocrine cell line as revealed by STED microscopy [Adapted by permission from Macmillan Publishers Ltd: Nature Communications (Saka et al., 2014), copyright 2015]. Scale bar: 500 nm.

More recently, a concept of **protein islands** was presented based on the fact that proteins were detected in distinct domains interspaced with protein-free areas, when membrane patches were imaged by electron microscopy (Wilson et al., 2000; Lillemeier et al., 2006). Heterogeneous distribution of proteins in entities reminiscent of such “protein islands” were often found by super-resolution fluorescence imaging of the cellular plasma membrane (for example Sieber et al., 2007; Lillemeier et al., 2010; Letschert et al., 2014; Saka et al., 2014; see also Figure 4). However, it is not yet clear whether such entities are created and stabilized by protein-protein interactions or other mechanisms are involved. The impact of the underlying actin cytoskeleton on protein islands was reported in the past (Wilson et al., 2001; Lillemeier et al., 2006).

Certain cytosolic proteins can **interact with headgroups** **of selected lipid species** (e.g., negatively charged phosphatidylserines and phosphoinositols) via electrostatic interactions (Figure 2C; McLaughlin and Murray, 2005). In cells, binding of proteins to charged headgroups of inner leaflet lipids is well-documented to control important cellular processes, e.g., phagocytosis (Botelho et al., 2000). In addition, peripheral protein interactions at the inner leaflet of the plasma membrane modulate localization and mobility of charged lipids, as well as some other, probably associated, molecules (Golebiewska et al., 2008, 2011; Yeung et al., 2008). But whether such peripheral interactions can modulate the mobility of other membrane components (e.g., at the outer leaflet) and have a more general impact on the plasma membrane organization awaits its direct proof.

Due to membrane plasticity and flexibity, certain lipids with non-conical shape, e.g., lysophospholipids or phosphatidylethanolamines found also in cell membranes, can deform the planar structure of lipid bilayers by bending (Figure 3B), thereby changing its **curvature** (Šachl et al., 2011). Similar to protein-protein interactions, curvature is not a typical extrinsic factor. But in cells, highly curved membranes are prevalently generated by curvature-forming proteins (e.g., BAR-domain containing proteins) or cytoskeleton-induced mechanical forces (McMahon and Gallop, 2005; Mattila and Lappalainen, 2008). Both these processes are externally regulated and require energy and/or cofactors (Mima et al., 2008; Frolov et al., 2010). For example, processes of endo/exocytosis are initiated by protein-induced membrane bending and ATP/GTP hydrolysis (Vilmart-Seuwen et al., 1986; Hansen and Nichols, 2009; Stachowiak et al., 2013). The plasma membrane has the capacity to form specialized extensions with high curvature to accomplish some of its specific functions, e.g., the formation of microvilli in polarized cells for the efficient uptake of nutrients (Crawley et al., 2014), or of membrane nanotubes for the inter-cellular communication (Onfelt et al., 2004). In theory, curvature can modulate the distribution of membrane molecules (Bozic et al., 2006; Wu and Liang, 2014). Indeed, some proteins accumulate in curved or filamentous membranes in cells, but the mechanisms responsible for such diversity are probably based on targeted delivery of molecules to these specific structures and partial impermeability of the basal region of such membrane extensions, e.g., of cilia (Trimble and Grinstein, 2015). In model membranes, specific proteins undergo curvature-driven sorting while others do not (Hatzakis et al., 2009; Aimon et al., 2014; Quemeneur et al., 2014). Specific intermolecular interactions between lipids and proteins were suggested to be responsible for such selectivity (Callan-Jones et al., 2011), nonetheless it is still unclear whether curvature-based protein and lipid sorting can occur in highly dynamic membranes of cells.

The **cortical actin** (CA) skeleton (Figure 3C) helps to keep and modulate the shape of living cells (Murase et al., 2004). In addition, it is involved in the regulation of membrane trafficking and signaling (for example Suzuki et al., 2007; Jaqaman et al., 2011; Gowrishankar et al., 2012; Johnson et al., 2012). The impact of the CA on the organization of the plasma membrane is well described and forms the basis of a key model discussed in the following section.

Similarly, the **glycocalyx** (Figure 3D), a part of the extracellular matrix (ECM) in vertebrates, forms a dense structure at the surface of eukaryotic cells (Stryer, 1995). Glycosaminoglycans and associated glycoproteins and proteoglycans of the glycocalyx were shown to modulate signaling by direct association with surface receptors (Bass et al., 2007; Morgan et al., 2007) or by binding of ligands (Hynes, 2009). In this way, the glycocalyx and the extracellular matrix regulate the shape of multicellular organisms. The glycocalyx was also predicted to influence the general organization of the plasma membrane (Jacobson et al., 1987). Indeed, glycosylated extracellular domains were shown to modulate the organization (Anderson and Fambrough, 1983) and mobility (Wier and Edidin, 1986; Zhang et al., 1991; Hartel et al., 2015) of membrane proteins. The molecular mechanism is still unknown and, to our knowledge, has not been studied in detail.

Cells keep their membranes “healthy” by a rapid turnover of its components. This is achieved mainly by vesicular transport—**endo-/exocytosis** (Figure 3E)—but also by a less well understood protein-mediated lipid transport mechanism(s) (Lev, 2012). Each exo-/endocytic event delivers or removes a material equivalent to a surface area of ≈ 30.000 nm^2^ (estimated for the average diameter of exo-/endocytic vesicles to be ~100 nm). Therefore, every such event can transiently, but dramatically change the local membrane composition and, thereby, organization. Whereas no preferred sites of exo-/endocytosis were reported under resting conditions (Schmoranzer et al., 2000), stimulation of cells can result in more localized vesicular transport and fusion/fission hotspots (Stinchcombe et al., 2001; Gaffield et al., 2009). This can further accelerate changes in the plasma membrane.

Alternatively, membrane components (specifically lipids) can be delivered to the plasma membrane by **specific lipid transporters** (Raychaudhuri et al., 2006; Voelker, 2009; Tarling et al., 2013). These may travel through the cytosol by diffusion or, more probably, such events can take place at **membrane contact sites** between the endoplasmic reticulum (ER) and plasma membrane (see Figure 5 in Fernández-Busnadiego et al., 2015). These sites are responsible for the synthesis, transport of lipids between the ER and plasma membrane (e.g., by Osh sterol transporters Raychaudhuri et al., 2006) and regulation of lipid metabolism in the plasma membrane (Stefan et al., 2011).

Vesicular and protein-mediated transport are the two main mechanisms responsible for a rapid turnover of membrane molecules (estimated to exchange almost all of its components within 1 h), but other routes such as free diffusion of small molecules (e.g., glucose, ions Cortizo et al., 1990) or infection of cells by viruses and other pathogens (Mazzon and Mercer, 2014) can further modulate membrane composition and organization.

Active transport of protons and ions, together with chemical asymmetry, generates an electrostatic potential across the plasma membrane of living cells (Hodgkin and Huxley, 1952). In addition to the function of the **membrane potential** in metabolism and transport of essential molecules in and out of cells, it has an impact on properties of model and cell membranes (O'shea et al., 1984; Grossmann et al., 2007; Herman et al., 2015). Even in the absence of ions, asymmetric distribution of lipids in the bilayer can generate a transmembrane potential (Gurtovenko and Vattulainen, 2007). As a consequence, it is technically challenging to uncouple membrane potential and asymmetry. Of note, the available theoretical and experimental evidence related to the electrostatic potential and the organization of cell membranes was recently reviewed (Malinsky et al., 2016).

On their own, extrinsic factors do not have the capability to control all plasma membrane processes. Hence, more holistic hypotheses combining intrinsic and extrinsic factors are needed. In the following section, we will briefly describe a more general concept and five most popular models. A reader will find more detailed descriptions of these models and some alternative views in recently published reviews (e.g., Lingwood and Simons, 2010; Owen et al., 2010; Klammt and Lillemeier, 2012; Klotzsch and Schütz, 2013; Nicolson, 2014; Rao and Mayor, 2014; Mouritsen and Bagatolli, 2015; Sevcsik and Schütz, 2016).

## Plasma membrane organisation–general models and concepts

Let us begin this section with a brief inspection of the mobility of membrane components. This will indicate how simple concepts highlighting intrinsic properties, namely viscosity and continuity can, to some extent, explain certain puzzles related to the plasma membrane organization and function. Measurements of lateral diffusion of membrane components over the last few decades uncovered much slower molecular mobility of molecules in cell membranes compared to their model counterparts (Wier and Edidin, 1986; Jacobson et al., 1987; Lippincott-Schwartz et al., 2001). On average, lipid tracers (e.g., DiI or BODIPY-DPPE) diffuse about four times faster in model membranes than in the plasma membrane of living cells (Box 1; Table 1). This difference can be explained by the compositional complexity of the plasma membrane. The large proportion of lipids with long and saturated acyl chains and cholesterol (van Meer et al., 2008) cause a higher rigidity (Sezgin et al., 2015) and, thereby, viscosity of membranes (Kucik et al., 1999). In addition, the presence of integral membrane proteins further increases the local viscosity in their immediate environment, which reduces the mobility of membrane constituents in general (Peters and Cherry, 1982; Chazotte and Hackenbrock, 1988; Frick et al., 2007; Saxton, 2008; Niemelä et al., 2010). A plethora of lipid-lipid and lipid-protein interactions, heterogeneities in general, can further contribute to this reduction in mobility.

**Table 1:** **Examples of diffusion coefficients and their translation to the times needed to traverse a distance of 20 μm (e.g., HeLa cell)**.

**Molecule and environment**	**Diffusion coefficient (μm^2^/s)**	**Time to traverse 20 μm (Brownian diffusion; seconds)^#^**	Reference(s)^*^
Small molecule (fluorescein) in water	430	0.2	Culbertson et al., 2002
Protein (GFP) in water	90	>1	Swaminathan et al., 1997
Small molecule (fluorescein) in cytoplasm	30	>3	Luby-Phelps et al., 1986
Protein (GFP) in cytoplasm (CHO cell)	30	>3	Swaminathan et al., 1997
Protein (GFP) in cytoplasm (bacterium)	8	12.5	Elowitz et al., 1999
Lipid tracer in fluid model membranes (DOPC; free-standing membrane)	5–15	1.6–20	Ramadurai et al., 2009
Lipid tracer in membrane blebs (cell membrane without cortical actin)	1–10	10–100	Tank et al., 1982
Lipid-anchored protein^@^ in fluid model membrane	5	20	Kahya et al., 2005
Integral membrane protein in fluid model membrane	2–5	20–50	Ramadurai et al., 2009
Lipid tracer in cell membrane	0.5–4	25–200	Tank et al., 1982
Lipid-anchored protein^@^ in cell membrane	0.1–1	100–1000	Zhang et al., 1991
Lipid-anchored protein^@^ in cell membrane blebs (without CA skeleton)	0.3–0.6	170–330	Zhang et al., 1991
Integral membrane protein in cell membrane blebs (CA skeleton-free)	0.01–0.5	200–10000	Tank et al., 1982
Integral membrane protein in cell membrane^$^	0.001–0.1	1000–100000	Tank et al., 1982

# *The time to traverse the distance x was calculated as τ ≈ x^2^/4D, where D denotes the diffusion coefficient*.

$ *Some membrane proteins can exhibit only small mobile fraction or have even slower D*.

@ *GPI-anchored proteins were tested in cited works*.

* *Original articles listed only*.

Therefore, intrinsic properties, particularly viscosity, can be responsible for the reduced long-range diffusion rates measured for lipids in cell membranes. Since membranes are continuous, all of its lipid components should be influenced similarly and equally throughout the whole area. For lipids which do not comply with this statement, localization and mobility is regulated by other factors such as proteins interacting with charged lipid headgroups, endocytosis,…etc. This simple concept works for lipids. But the extremely slow mobility of many plasma membrane proteins—one-to-two orders of magnitude lower compared to model membranes–calls for a more elaborate explanation.

**Figure 5:**
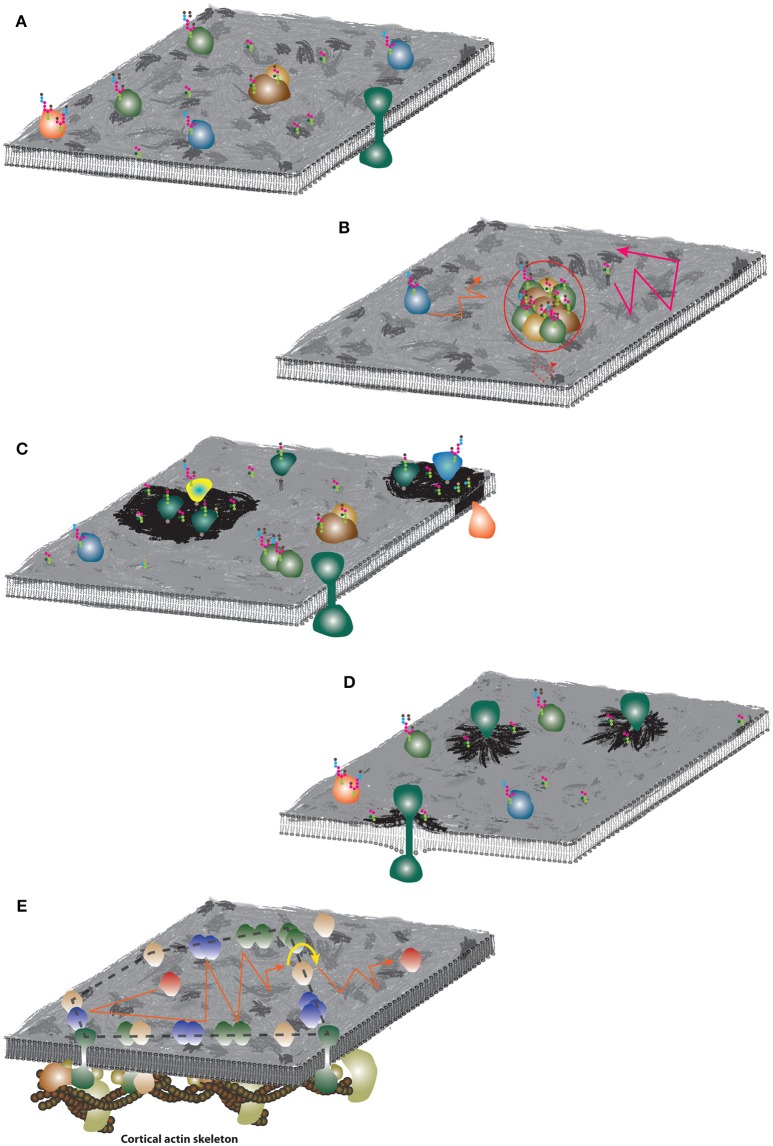
**Schematic illustrations of the plasma membrane organization models. (A)** Fluid mosaic model. The membrane surface was artistically decorated to indicate non-homogenous distribution of molecules. Colored objects represent various species of membrane proteins, strings of colored hexagons illustrate glycosylation of proteins and lipids. **(B)** Hydrodynamic model. Similar mobility of lipids and proteins are indicated by orange and pink trajectories. Large assemblies (red circle) with significantly larger radius can exhibit slower diffusion (dashed red trajectory). **(C)** Lipid membrane domains. Dark membrane patches indicate lipid self-assemblies and different lipid (and protein) composition. **(D)** Mattress model. Dark membrane patches indicate accumulation of lipid species due to increased hydrophobic length of protein TMDs. **(E)** Picket-and-fence model. Accumulation of proteins around the underlying CA skeleton and formation of fences (dashed black line) which may restrict “free” diffusion of non-associated proteins (red) to a limited area (red trajectory). For long-distance mobility, proteins have to “hop” over the fence (yellow arrow-line) which limits their long-range diffusion coefficient.

*Fluid Mosaic Model (SN Model; Figure* *5A**)*. The SN model in a large detail summarizes the understanding of the plasma membrane composition, structure and thermodynamics 45 years ago (Singer and Nicolson, 1972). The emphasis is placed on the fluidity of the membrane and coexistence of lipids and proteins in this essential cellular structures. We have already described crucial issues of this model in the previous sections. Here, we would like to underline that the word “mosaic” in the SN model was primarily used to accent a mixed character of cell membranes where diverse lipids and proteins coexist in a single lamellar structure. Later, this was frequently misinterpreted as homogeneous or random distribution of molecules. But **heterogeneity** of cell membranes was observed and reported as early as in 1960s (Figures 4A,B; Cerottini and Brunner, 1967; Aoki et al., 1969; Kourilsky et al., 1971; Nicolson et al., 1971). Indeed, Nicolson described putative mechanisms responsible for clustering of proteins (or formation of domains) in his pillar work already in 1979 (Nicolson, 1979 and Figure 4 therein). These assumptions are still valid almost 40 years later (Nicolson, 2014).
*Hydrodynamic Model (Figure* *5B**)*. The mobility of transmembrane proteins and their aggregates in cell membranes can be defined by the hydrodynamic model (Saffman and Delbrück, 1975). This model hypothesizes that molecular diffusion rates depend mainly on membrane viscosity and thickness, and only weakly on the size of proteins and aggregates. This model was later updated many times (e.g., for arbitrary viscosity of membranes and solutes (Hughes et al., 1981) or asymmetric membranes Evans and Sackmann, 1988), and experimentally confirmed in model membranes (e.g., Ramadurai et al., 2009). However, it applies only for freely moving molecules absent of interactions with objects which do not co-diffuse as a single entity. The model is further limited by the density of objects in the membrane and their lipid environment. First, the presence of slowly moving obstacles and molecular crowding can strongly influence the mobility of membrane components (Saxton, 2008; Guigas and Weiss, 2015). Second, lipids in the vicinity of TMDs of integral membrane proteins (annular lipids or lipid shells) exhibit reduced lateral diffusion (Meier et al., 1987; Anderson and Jacobson, 2002). This is probably caused by the fact that TMDs form relatively large and rigid structure in the bilayer (Meier et al., 1987; Niemelä et al., 2010) but also due to the rough surface of TMDs. Therefore, the complexity of cell membranes evidently does not allow the application of hydrodynamic model or its variants as a general model of the plasma membrane organization. Nevertheless, it can provide a useful alternative to more complex models for local changes (nanoscale; see below).
*Self-Assemblies of Lipids and Ordered Lipid Domains (Figure* *5C**)*. Observation of protein clusters (DePierre and Karnovsky, 1973), lipid segregation (Shimshick and McConnell, 1973a,b; Klausner et al., 1980) and heterogeneous distribution of certain lipids and proteins between apical and basal membranes of polarized cells (van Meer and Simons, 1982) led to the suggestions that lipids and their self-assemblies can determine the fate of newly synthesized or recycled membrane molecules (Karnovsky et al., 1982; Simons and van Meer, 1988). This concept was modified by Simons and Ikonen (Simons and Ikonen, 1997) who proposed “lipid rafts” as the plasma membrane platforms of high molecular order enriched in cholesterol and sphingolipids, in which proteins involved in signaling can selectively interact with effector molecules. In parallel, biochemical analyses revealed inefficient solubilisation of some, but not all, membrane proteins and lipids in mild detergents, forming the basis of detergent resistant membranes (DRMs). Throughout the years, the ordered lipid character of “model lipid rafts” was emphasized and suggested to correspond to domains present in the plasma membrane of cells. All these terms, lipid rafts, DRMs and ordered lipid domains, were used inconsistently and frequently led to misinterpretations which were highlighted in recent reviews (Cebecauer et al., 2009; Owen et al., 2010; Kraft, 2013; Sevcsik and Schütz, 2016). In addition, the data supporting spontaneous formation of lipid domains in living cells are rather controversial and inconclusive (e.g., Eggeling et al., 2009; Brameshuber et al., 2010; Owen et al., 2012; Honigmann et al., 2014; Sevcsik et al., 2015). On the other hand, an undisputable capacity of certain lipids (e.g., gangliosides) to self-aggregate (Fujita et al., 2007; Chen et al., 2008), anomalous diffusion and/or distribution of lipids in highly complex mixtures (Kusumi et al., 2005; Eggeling et al., 2009; He and Marguet, 2011; Jeon et al., 2012) and spontaneous formation of fluid nanoclusters (van Zanten et al., 2010; Amaro et al., 2016) were demonstrated *in silico*, in model membranes as well as in living cells. Such fluctuations can potentially contribute to the overall heterogeneity of the plasma membrane and the peculiar mobility of certain lipids and proteins therein. Yet, the direct observation of such anomaly remains challenging due the required spatial and temporal resolution to disclose molecular-scale objects at sub-millisecond rates, albeit recent advances in super-resolution optical microscopy and ultrafast single-molecule tracking indicate remedies to this limitation.
*Mattress Model (Figure* *5D**)*. As mentioned above, lipids in the vicinity of TMDs exhibit abnormal behavior (Lee, 2004; Niemelä et al., 2010), particularly in cell membranes with a large variety of lipid species and TMDs. The average membrane hydrophobic thickness increases between the ER, Golgi apparatus and plasma membrane (Mitra et al., 2004). During protein translation, proteins with long TMDs are incorporated into the relatively thin membrane of the ER, causing hydrophobic mismatch. Lipids with longer and saturated acyl chains can form metastable shells surrounding such TMDs, thereby generating heterogeneity in the membrane of the ER. At a larger scale, hydrophobic mismatch was proposed to induce the formation of lipid/protein domains also in the plasma membrane (Mouritsen and Bloom, 1993; Anderson and Jacobson, 2002; Kaiser et al., 2011). Significant impact of hydrophobic mismatch is well-documented for the sorting of proteins in cell membranes (Munro, 1995; Sharpe et al., 2010; Chum et al., 2016). But whether similar “sorting” of lipids and proteins due to hydrophobic mismatch contributes to the nanoscale organization of the plasma membrane in living cells has so far not been experimentally proven, mainly due to aforementioned limitations on spatial and temporal resolution of potential direct observation methods.
*Cortical Actin Skeleton (Figure* *5E**)*. Membrane-proximal positioning of the CA skeleton and its direct association with the plasma membrane via actin-binding proteins or complexes makes it the first-hand structure to influence the mobility of plasma membrane molecules and their lateral organization. Indeed, the actin skeleton was demonstrated to affect membrane molecules in numerous works employing a variety of experimental approaches (e.g., Golan and Veatch, 1980; Sheetz et al., 1980; Tank et al., 1982; Fujiwara et al., 2002; Ritchie et al., 2003; Murase et al., 2004; Mueller et al., 2011; Andrade et al., 2015). The effect of the CA skeleton is to date the most accepted model for membrane organization, independent of whether we speak about indirect sterical hindrance (picket-and-fence model; (Koppel et al., 1981; Jacobson et al., 1984; Sako and Kusumi, 1995; Machta et al., 2011) or direct interactions of proteins with the CA skeleton (Saxton, 1990; Sheetz et al., 2006; Mueller et al., 2011; Rao and Mayor, 2014). Its undisputable impact was described in more detail in current reviews (Kusumi et al., 2010; Rao and Mayor, 2014). On the other hand, the CA skeleton provides a good explanation for many, but probably not all membrane-associated phenomena (see below).

## There is no universal model of the plasma membrane lateral organisation

Models listed in the previous sections, better or worse, contribute to the overall understanding how cells potentially organize molecules in their plasma membrane. Some of these models passed through their glorious periods, in which almost any article assumed the applicability of this one particular mechanism for the function and/or organization of the studied membrane molecule(s). A handful of recent experimental work (e.g., Kenworthy et al., 2004; Frisz et al., 2013; Honigmann et al., 2014; Letschert et al., 2014; Sevcsik et al., 2015; Wilson et al., 2015) and reviews (Kraft, 2013; Sevcsik and Schütz, 2016) argue against these universal theories. Improvements in technology for observing membrane studies have more and more reduced the affection for such a single, universal theory. A dynamic and complex plasma membrane is the environment where all molecules play in concert to achieve the optimal physiological output.

As a metaphor, one can think of human society. Similar to cell membranes, it is highly complex and dynamic, with activities difficult to investigate. As an example one can consider clustering. “Clustering” occurs in human society at the nanoscale (e.g., families), mesoscale (e.g., clubs, classes or other small interest groups), or macroscale (e.g., villages, cities, states). The formation of such “clusters” depends on intrinsic properties like affection or animosity, the local or global economic situation, but also the health and mobility of the individuals. As an analogy for extrinsic parameters we may consider the environmental situation (sunshine/rain, drought/flooding), local factors (alpine landscape vs. influence of the sea), but also the interaction with other “clusters.” As we know from experience, social systems may develop rather stable phenotypes at the macroscale (e.g., the current western society), which are still characterized by high dynamics at the nano—or mesoscale. On the contrary, there are periods in history, in which no stable situation was reached for many years. Our point is, that it is virtually impossible to predict the behavior of a large society from simple models, even if the intrinsic and extrinsic parameters are well-known at high detail. Or, if we return to the topic of cell membranes: currently, it seems impossible to explain the plasma membrane organization based on individual models described in the previous section. Hence, future challenges will include the clever combination of this principle models into more holistic meta-models to increase their predictive power. Or, in the other words, we believe there is no simple, universal mechanism underlying the organization of the plasma membrane of mammalian cells.

Why we believe this is so? And what are the consequences?

Starting with the first question, one has to look at the sections with the lists of intrinsic and extrinsic factors influencing the behavior of molecules in the plasma membrane. Both, intrinsic and extrinsic factors are highly interconnected and can occur at the same time or, more probably, in rapid, sequential events. If intrinsic properties should be considered as rather general factors, to which all molecules must adapt, extrinsic factors may have more specific effects. Tuning of intrinsic properties (e.g., fluidity or viscosity) requires significant changes in molecular composition. This can rapidly occur locally (at the nanoscale) and transiently (sub-second), but would require substantial costs of energy to induce large-scale and more stable changes. On the contrary, extrinsic factors (e.g., the CA skeleton or glycocalyx) can affect larger surface areas for longer periods of time with higher efficiency. It is, therefore, probably a combination of these factors which regulates behavior of molecules in the plasma membrane at a full spectrum of spatial and temporal scales.

This brings us to the second question about the consequences of the non-existence of omnipotent, universal model applicable to all plasma membrane components and events. First, when interpreting data acquired during the analysis of cell membranes, one should not ignore intrinsic membrane properties. Even though less visible (detectable), these form the basis of membrane organization and function. Extrinsic factors are important but may be consequential. In order to fully understand membrane-associated processes and avoid undesirable borders of a single theory, a careful analysis of sequential events, which may lead to the observed effect, needs to be performed (Box 2).

Box 2 **Sequential events influencing the plasma membrane organisation**

Imaging techniques are excellent tools to monitor changes of the plasma membrane organization. High details can be explored using current advanced techniques (Eggeling, 2015). But all methods suffer from the fact that the preeminent feature (e.g., CA skeleton reorganization) can hide one or more less well detectable events (e.g., changes in local viscosity) accompanying an observed process. In some cases, these undetectable fluctuations may be the determining factors or triggers of a transformation process.

To illustrate the consequences of the abovementioned limitation(s), let's imagine a putative membrane-associated process: A ligand binds to its receptor which is followed by receptor oligomerisation or nanoscale clustering. Such increased protein density causes increased membrane viscosity which, in turn, reduces mobility of molecules in the vicinity of a cluster (Peters and Cherry, 1982; Niemelä et al., 2010). As a consequence, an actin-binding protein can collide with a receptor cluster, enhance low-affinity interactions by crosslinking cluster components and trigger CA skeleton reorganization. It is the last event which will stabilize the overall structure and, at the same time, it is the best detectable feature of this imaginary process. But the interpretation that the CA skeleton is responsible for the observed changes is only part of the story. In this case, the ability to detect small-scale viscosity fluctuations would help to better understand such process. Unfortunately, at present, such tools are not available for living cells.

Another concern with the interpretation of membrane-focused data is the systemic use of chemical and genetic tools as a proof of one or the other model of the plasma membrane organization. Specific side-effects of some of these treatments (e.g., detergents, methyl-β-cyclodextrin, cytochalasin D or temperature changes) have been described in past (Ailenberg and Silverman, 2003; Lichtenberg et al., 2005; Magee et al., 2005; Shvartsman et al., 2006; Zidovetzki and Levitan, 2007). Due to the fluidity and cooperativity, systemic treatment (both, chemical and genetic) will often influence the behavior of many (if not all) molecules present in or associated with the membrane, instead of only specific ones. In addition, the procedure of observing the system may potentially introduce artifacts, for example labels or intense light sources employed in fluorescence microscopy (Sezgin et al., 2012; Magidson and Khodjakov, 2013). Therefore, employment of treatments or observation techniques requires cautious interpretation and experiments performed with extensive number of controls. Leaving space for alternative interpretations and emphasis on possible side-effects should be a good practice in this kind of works.

In summary, we provide here a comprehensive list of membrane features and peripheral structures which were previously demonstrated or proposed to control lateral mobility and organization of the plasma membrane in mammalian cells. We also offer alternative views how to interpret results measured on the plasma membrane of living cells. We re-emphasize the impact of the intrinsic membrane properties which were discovered and characterized more than 20 years ago but were sometimes overlooked in more recent works. We finish with the hope that development of novel improved observation techniques such as fast single-molecule tracking (Ritchie et al., 2005; Ortega-Arroyo and Kukura, 2012), TOCSSL (Brameshuber et al., 2010), STED-FCS (Eggeling et al., 2009; Mueller et al., 2013; Eggeling, 2015), iMSD or related image correlation techniques (Hebert et al., 2005; Digman et al., 2009; Di Rienzo et al., 2013), will be rewarded with a more precise information about players responsible for the uniqueness of the plasma membrane. In case the improvements will be still insufficient, we should probably overpass the barrier (obstacle) between researchers studying mammalian cells and those focused on yeasts and plants. These organisms own membranes which behave much friendlier on temporal scale compared to the plasma membrane of mammalian cells. Such membranes are highly heterogeneous and can be imaged with the use of existing methods (Malínská et al., 2003; Spira et al., 2012). Cell cycle regulation and RNA interference were also discovered in yeast and plants.

## Author contributions

JBdlS, GS, CE, and MC defined the topic and wrote the manuscript.

## Funding

This work was funded by Czech Science Foundation (15-06989S; MC), the Medical Research Council (MRC, grant number MC_UU_12010/unit programmes G0902418 and MC_UU_12025; CE), Marie Curie Career Integration Grant (JBdlS), and the Austrian Science Fund (FWF projects P 26337-B21, P 25730-B21).

### Conflict of interest statement

The authors declare that the research was conducted in the absence of any commercial or financial relationships that could be construed as a potential conflict of interest.
